# A social science research agenda to accelerate public engagement in climate change adaptation

**DOI:** 10.3389/fpsyg.2023.1286525

**Published:** 2023-12-04

**Authors:** Rachel Harcourt, Suraje Dessai, Wandi Bruine de Bruin, Andrea Taylor

**Affiliations:** ^1^Centre for Decision Research, Leeds University Business School, University of Leeds, Leeds, United Kingdom; ^2^ESRC Centre for Climate Change Economics and Policy, Sustainability Research Institute, School of Earth and Environment, University of Leeds, Leeds, United Kingdom; ^3^Priestley Centre for Climate Futures, University of Leeds, Leeds, United Kingdom; ^4^Sol Price School of Public Policy and Dornsife Department of Psychology, University of Southern California, Los Angeles, CA, United States

**Keywords:** climate change adaptation, public perceptions, research agenda, communications, engagement, social science, action

## Abstract

Recent studies find that people in high-income countries now think of climate change impacts, such as flooding or periods of high temperature, as being of immediate relevance and concern. Individuals and households can take adaptation actions to help limit the severity of harm caused when climate impacts occur, for example, they could make adjustments to their homes such as installing flood gates or sun shades, or they could adapt their behavior such as staying indoors during the hottest part of the day. However, so far adaptation is not yet happening at the speed or scale needed to adequately prepare for the climate impacts already being experienced or those projected for the coming decades. Here, we propose an agenda for future social science research that would further our understanding of how best to increase engagement and action in climate change adaptation.

## Introduction

Until recently surveys in high-income countries tended to find that people thought of climate risks as mainly a concern for other parts of the world and for future generations (Leiserowitz, 2006; Lorenzoni and Pidgeon, 2006). However, in the last several years there has been a shift, with people increasingly reporting that they think climate change risks are ‘here and now’, that they are worried about them, and that they think comprehensive and immediate action needs to be taken (e.g., IPSOS, 2020; Steentjes et al., 2020; UNDP and University of Oxford, 2021; Lloyd’s Register Foundation and Gallup, 2022). The likelihood and severity of harm caused by climate impacts can be, in part, ameliorated by climate change adaptation actions, such as building sea walls to reduce the likelihood of storm surges causing flooding and adding water resistant features to buildings to reduce the severity of the damage if they are breached by flood water. And yet a recent global stocktake found that so far adaptation efforts are “largely fragmented, local and incremental with… negligible evidence of risk reduction outcomes” (Berrang-Ford et al., 2021, p.989). That people in high-income countries now see climate risks as personally relevant and serious might be the catalyst needed to greatly accelerate and expand adaptation action. Below, we outline a research agenda to support that ambition. We recognize the topic is vast and all possible avenues cannot be captured in this short Review; we therefore focus on areas in which we have greatest collective expertise, namely public perceptions, communications and engagement.

## Research topic 1: How are people’s perceptions of climate risks changing?

While, as summarized above, there has been notable agreement regarding perceptions of climate risks in recent surveys, the context in which people’s opinions are formed is constantly changing. As such, understanding of public perceptions of climate risks needs to be kept up-to-date. In many parts of the world 2022 was an exceptional year for extreme weather, including the European heatwaves from June to September, Hurricane Ian causing extensive damage to parts of the Caribbean and East Coast USA, and the devastating Pakistani floods. So far in 2023 much of the northern hemisphere has experienced further record-breaking heatwaves and wildfires while parts of Asia and the USA have had serious flooding. Research could examine whether these ongoing severe weather events increase climate change concerns, or whether the ‘shifting baselines syndrome’ applies to experience of extreme weather events, as has been found with other environmental concerns, such as falling numbers of wildlife (Soga and Gaston, 2018). As some events, such as summer heatwaves in traditionally cooler climates, become more frequent they might come to be seen as ‘normal’ therefore reducing people’s perception of them as a risk that requires action. In recent years, populations have also experienced the continuation of the Covid-19 pandemic, the outbreak of a land war in Europe, and a cost-of-living crisis. Research could also provide understanding on whether some events have more influence on perceptions of climate risks than others. While a majority of people around the world are reporting concerns about climate change, only 3% mentioned climate change or severe weather among their top concerns (Lloyd’s Register Foundation and Gallup, 2022). Additionally, studies found that while the Covid-19 pandemic did not lessen people’s climate concerns (e.g., UNDP and University of Oxford, 2021; Lloyd’s Register Foundation and Gallup, 2022), the 2007–2009 Great Recession did (Scruggs and Benegal, 2012; Shum, 2012). With this in mind, research should test whether highlighting the links between climate change and other risks of concern affect willingness to implement climate change adaptation. Such insights can ensure that those tasked with engaging people on managing climate risks are aware of the wider context of people’s perceptions and can tailor their outputs accordingly.

## Research topic 2: How do people perceive climate change adaptation?

Despite the urgent need for governments to significantly accelerate their nation’s preparedness for climate impacts (Berrang-Ford et al., 2021), there has so far been limited research into perceptions of climate change adaptation with populations of high-income countries. There are some exceptions such as a large, multi-part study in the UK in 2012-13 which found generally strong public support for adaptation (IPSOS MORI, 2013). However, considering the recent pace of change in climate risk perceptions there’s a need to both update these studies and add much more detail. For example, when do people, particularly in countries which have not until recently experienced many instances of extreme weather, think adaptation should happen? Are they willing to prioritize public spending on this issue over others? What types of adaptation policies do people support? What level of risk do they consider acceptable versus what level of adaptation? These are some of the questions that might be asked to get a better understanding of public perceptions of climate adaptation and, crucially, public support for it.

In 2022, England ran its first adaptation-focused public dialogue with 112 participants recruited to deliberate what being ‘well-adapted’ to climate change should look like. The participants strongly prioritized human well-being and safety particularly for the most vulnerable, and hoped for a future population which is well educated on the topic in a country that is prepared and flexible to change (Brisley et al., 2023). This type of research could be replicated elsewhere using public dialogues or other forms of participatory research such as citizens assemblies. Once a vision of ‘well-adapted’ is established, research would also need to identify pathways to achieving it including consideration of the trade-offs and instances of loss it might entail. There are some few studies into public perceptions of adaptation policies which seem to suggest growing levels of public support over time (e.g., comparing the low levels of public support for a list of suggested adaptation policies reported in Hagen et al. (2016) compared with the generally higher support reported in van Valkengoed et al. (2022)). However, given the scale of government-led adaptation action required over the coming years to achieve at least adequate levels of resilience, research in this area needs to be much more robust. Studies can be undertaken at a scale aligned with decision making, ranging for example from in-depth research with coastal communities planning local resilience to projected sea level rise to national studies of willingness to invest public money in adaptation compared to other public services, and should be used to inform local, regional and national adaptation program and policies.

## Research topic 3: Why are people not acting despite being concerned?

While concern about climate change is now high, it is not yet leading people to take their own adaptive actions, such as increasing the resilience of their homes to extreme weather, commensurate with the risks they face now and in the coming decades (e.g., Power et al., 2020). One potential barrier is that people do not necessarily know what ‘climate change adaptation’ is (Harcourt et al., 2019; Bruine de Bruin et al., 2021). Some have argued that conservative audiences may be more willing to implement climate change adaptation policies against, say, flooding, if climate change is not mentioned, in part because changes in extreme weather are a more concrete experience than that of climate change (Bruine de Bruin et al., 2014). Additionally, some barriers will be context specific, for example, a review of UK household adaptation found that other barriers to implementing adaptation actions included cost, concerns about disruption, and unwanted changes to the look of the property (Porter et al., 2014). Policies such as grants for households and a rapidly evolving commercial market for adaptation could help to address such barriers. However, there is also some evidence of the influence of social barriers (Adger et al., 2009; Biesbroek et al., 2013). For example, people may be less likely to act if they feel that they are being asked to take on more responsibility than other individuals or groups (Gifford, 2011). Here, social science can add useful insight into how these social barriers are experienced in differing contexts and how they can be overcome. In some instances, a perceived barrier, such as a sense that adaptive action is not well accepted by an individual’s peer group, could become a motivator, if adaptive action comes to be seen as a positive social norm (Mildenberger and Tingley, 2017).

As such, we need to more clearly understand what motivates people to take personal adaptive actions and to partake in local and community initiatives (Berrang-Ford et al., 2021). A recent meta-analysis of the motivational factors most associated with adaptive behavior found that research has so far focused on a limited number of hazards, motivational factors, and adaptive behaviors, resulting in an incomplete picture (van Valkengoed and Steg, 2019). Nevertheless, there is work to build on. The meta-analysis identified perceived self-efficacy, outcome-efficacy, and the perception that others are taking similar actions as likely motivators (van Valkengoed and Steg, 2019). Other possible motivators include the perception that adaptive action is morally the right thing to do (Adger et al., 2017), that adaptation can protect things most of value (Harcourt et al., 2019), and is well aligned with pre-existing social identities (Barnett et al., 2021), and that individuals have a responsibility to adapt (Cotton and Stevens, 2019). Research can test the robustness of these motivators with different individuals and communities under different scenarios, and further add to the list. Research also then needs to consider how effective motivators can be best engendered in diverse populations.

Adaptation action can also be motivated by interventions, often government-led. At the individual and household scale, interventions to encourage the mitigation of greenhouse gasses and to encourage greater sustainability are numerous and well established. For example, to name just a couple, many countries give tax reductions to those driving low emitting cars, and require households to separate their recyclable waste from landfill, using either financial incentives or the risk of a fine to achieve compliance. For adaptation, regulations are being used in the business sector to motivate greater understanding of organizations own risk exposure and to develop resilience. For example, since 2022, reporting climate exposure using the Task Force on Climate-Related Financial Disclosures (TCFD) structure has been mandatory for large businesses in the UK and is increasingly being adopted in other countries. However, there are so far few society scale interventions to motivate individual adaptive action, perhaps in large part because adaptation tends to be very context specific (Biesbroek et al., 2013). In other words, the adaptive actions which would benefit high income households in rural areas at risk of river flooding might be different to those which would benefit low income households in cities at risk of urban heat stress, making it difficult to develop interventions with broad relevance and usefulness. Nevertheless, research can help move understanding of this forward both by testing proposed interventions in experimental studies and by reporting on the effectiveness of any that do appear within society such as financial support for home retrofitting.

## Research topic 4: How best to develop communications that motivate action?

Public communications can have a key role to play in motivating greater adaptive action. Much communication has so far focused on convincing people that climate change is real and serious. The recent shift in reported public opinion suggests that message has now landed, albeit we recognize that increased personal experience of extreme weather events may also be a significant factor in people’s increasing perceptions of risk (Bruine de Bruin and Dugan, 2022). That signifies a need to now focus on communicating how people can respond to the risks, including how to evaluate personal climate risk, what people can do to manage their own risk, and information on adaptation policies. The process of developing, evaluating, improving, and disseminating these communications needs to be accelerated. Communications can also support the motivating factors developed under the previous topic. For example, the recent survey findings summarized at the beginning of this article suggest an opportunity for public communications to emphasize the extent to which concern about current and local climate change risks is now widely shared. This is important because the (mis-) perception that society has lower levels of climate change concern can be one factor limiting an individual’s willingness to commit themselves to pro-climate positions (Mildenberger and Tingley, 2017). In contrast, there is evidence that perceiving action as something that others are also doing is a principal motivator of adaptation action (van Valkengoed and Steg, 2019; Power et al., 2020) and that a perceived consensus regarding climate change can be a causal factor in increasing public support for climate policy (van der Linden et al., 2015). Therefore, communications should emphasize the consensus of opinion and in time aim to establish it as a social norm.

However, while information-driven communications will remain important, their limitations as a means of engagement is well reported in the literature (e.g., Whitmarsh et al., 2021). In recent years, climate change research has seen an increased use of a broad spectrum of citizen participation approaches. For adaptation research this has taken many forms including public dialogues (Brisley et al., 2023), interactive art installations (e.g., Burke et al., 2018; Aragon et al., 2019) and futures thinking (e.g., Harcourt et al., 2021). Social science research has at least three significant contributions to make here. Firstly, it can add to the content and design of participatory research. For example, several of the authors of this paper contributed adaptation and public engagement expertise to the England’s recent public dialogue on the topic (Brisley et al., 2023). Secondly, researchers can collect and analyze data during the participation activities to understand how effective they are toward their stated goals, an element missing in much outreach work (Burke et al., 2018). Although it is often assumed that participatory approaches are more engaging, evaluative research can add a more critical view and learnings can help ensure that future funds and efforts are directed toward the most effective forms of engagement. Finally, one limitation to participatory research is that, due to the time and costs it often requires, sample sizes tend to be small. Social science research can contribute by testing the robustness of the findings with large samples through surveys or similar.

## Outlook

In this Mini Review, we have reflected on the trend in recent surveys that finds that people in high-income countries increasingly think of climate change impacts as a ‘here and now’ concern, and considered how public perceptions and engagement research might further build on this to accelerate and expand adaptation action. Yet, despite high levels of public awareness and concern about climate impacts, the most recent evidence suggests that public prioritization of climate change as an urgent issue for governments to address has fallen slightly, with public focus now on the cost of living crisis, the economy, and health and wellbeing (Harcourt et al., under review)^1^. Further, there are increasing signs that the developed world is experiencing an ‘anti-climate backlash’ as governments seek to back-peddle on policies perceived as costly and disruptive to voters (The Economist, 2023). This creates an even greater and more urgent challenge for the adaptation research community as it seeks to encourage governments and citizens to maintain, and indeed increase, momentum on preparing for the impacts of climate change, while swimming against these currents. Here, we have highlighted recent research which is starting to move beyond understanding perceptions of risk and toward engaging people in climate change adaptation, and we have outlined a number of fruitful approaches to be explored. Nevertheless, we recognize the challenge ahead and hope this Review may inspire thought and conversation as to where research can best contribute.

## Author contributions

RH: Conceptualization, Writing – original draft, Writing – review & editing. SD: Writing – review & editing. WBB: Writing – review & editing. AT: Writing – review & editing.

## Footnotes

^1^Harcourt, R., Sambrook, K., Dessai, S., and Taylor, A. A. (Under review). 10-year longitudinal analysis of public perceptions of climate risks and adaptation.
